# Benchmarking the nutrient composition and labelling practices of finger foods and snacks for older infants and young children across seven Southeast Asian countries

**DOI:** 10.1111/mcn.13598

**Published:** 2023-12-13

**Authors:** Alissa M. Pries, Eleonora Bassetti, Jessica M. White, Anzélle Mulder, Diane Threapleton, Jessica Blankenship

**Affiliations:** ^1^ Helen Keller International New York New York USA; ^2^ UNICEF East Asia Pacific Regional Office Bangkok Thailand; ^3^ JB Consultancy Johannesburg South Africa; ^4^ School of Food Science and Nutrition University of Leeds Leeds UK

**Keywords:** childhood diet, child public health, complementary feeding, complementary foods, infant and child nutrition, infant and young child feeding

## Abstract

Ensuring consumption of nutrient‐dense, safe and appropriate complementary foods among older infants and young children (IYC) 6–36 months of age is critical for enabling optimal growth and development. The ubiquitous availability of and high demand for commercially packaged snack foods has culminated in a growing trend of snack food products specifically produced and promoted for older IYC. Commercially produced complementary foods (CPCF) that are finger foods/snacks often contain added sugars, excessive sodium content and high total sugar content, making them inappropriate for this young population. This study benchmarked the nutrient composition and labelling practices of CPCF finger foods/snacks available for purchase in seven countries in Southeast Asia. The study adapted a nutrient profiling model from the WHO Regional Office for Europe to determine the proportion of products suitable for promotion for older IYC. Of the total 606 products identified, 8.2% were automatically categorized as not suitable because they were confectionery items. Of the remaining 556 products assessed, over 85% failed to meet all nutrient composition requirements, with the presence of added sugars/sweeteners and excessive sodium and total sugar contents the primary reasons for failure. Products also demonstrated concerning labelling practices, with all of the products (98.6%) displaying an inappropriate claim on the label. These findings reveal major concerns with the nutrient composition and labelling practices of CPCF finger foods/snacks in the SEA region and should serve as an alarm bell for regulatory action. National binding legal measures, such as mandatory standards for composition and labelling are urgently needed.

## Key messages

- CPCF finger foods/snacks made up 18‐45% of CPCF products across the seven countries, indicating a normalization of commercially packaged, processed snacks products in older IYC diets.
- Over 85% of products failed to meet all nutrient composition requirements. Three‐quarters contained added sugars/sweeteners, half high total sugar, and half excessive levels of sodium, indicating that most products are not nutritionally appropriate for this young age group.
- No CPCF finger foods/snacks met all labelling requirements and most displayed inappropriate claims. With nearly all products containing excessive levels of total sugar, added sugar or sodium, use of persuasive claims on these products is alarming.

## Graphical abstract

This study benchmarked the nutrient composition and labelling practices of 556 commercially produced complementary food  finger foods/snacks available in seven countries in Southeast Asia. The majority failed to meet all nutrient composition requirements and none met all labelling practice requirements, primarily due to the presence of added sugars/sweeteners, excessive sodium and inappropriate claims on the label; these findings serve as an alarm bell for regulatory action.

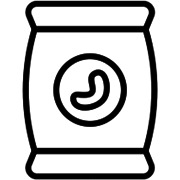

## 1 INTRODUCTION

The proliferation of highly‐processed, packaged foods within the global food system has expanded over the last several decades, with per capita sales of these foods increasing by 46% in Asia between 2005 and 2017 (Development Initiatives, 2018). This market growth reflects movement of food habits and choices towards less expensive packaged foods that cater to consumers' needs for convenient options and an inclination towards hyper‐palatability (Djupegot et al., 2017; Gibney et al., 2017; Machado et al., 2017). This shift in diets has not only been marked among adults and school‐age children, but also older infants and young children (IYC) from 6 months to 3 years of age (Pries, Filteau, et al., 2019). In various HIC and LMIC contexts, the consumption of commercially packaged snack foods promoted to the general population has been found to contribute 20% or more of energy intakes among children under 2 years of age (Karnopp et al., 2017; Kavle et al., 2015; Pries, Sharma, et al., 2019; Rodríguez‐Ramírez et al., 2016; Webb et al., 2006). Studies in the Southeast Asia region have noted consumption of commercially packaged snack food products among 82% and 55% of older IYC in urban Indonesia and Cambodia, respectively (Green et al., 2019; Pries et al., 2017). The ubiquitous availability of and high demand for commercially packaged snack foods has culminated in a growing trend of snack food products specifically produced and promoted for older IYC. An assessment of commercially produced complementary foods (CPCF) in the United Kingdom market found that the proportion of CPCF products that were finger foods/snacks increased from 10% in 2013 to 21% in 2019, growing from 42 to 185 products (Garcia et al., 2020), and Ireland has seen a 72% increase in the number of CPCF finger foods/snacks marketed between 2011 and 2017 (Geraghty et al., 2018). While CPCF finger foods/snacks make up one‐fifth of CPCF products in the British market, they account for approximately three‐fifths of the portions/servings purchased for infants and young children (Public Health England, 2019), with consumption of CPCF finger foods/snacks rising steadily with age (United Kingdom Department of Health, & United Kingdom Food Standards Agency, 2011). A recent study among Australian children 9–15 months of age found that children consumed CPCF finger foods/snacks almost daily and made up almost 10% of their total energy intake (Moore et al., 2022).

Ensuring consumption of nutrient‐dense, safe and appropriate complementary foods starting from 6 months of age, with the continuation of breastfeeding, is critical to facilitate adequate nutrition in the diets of older IYC and enable their optimal growth and development (Pan American Health Organization, & WHO, 2001; World Health Organization, 2005). While provision of snacks during the complementary feeding period is a global recommendation, the nutritional quality of these snacks is critical to ensure they contribute appropriately to older IYC energy and nutrient requirements (Pan American Health Organization, & WHO, 2001; World Health Organization, 2005). Due to limited gastric capacity, the nutritional quality of foods consumed during this age is vital and the World Health Organization (WHO) recommends the feeding of nutritious snacks, with most infants able to consume finger foods by approximately 8 months of age (Pan American Health Organization, & WHO, 2001). The use of CPCF in older IYC feeding is increasingly common, yet the nutritional quality and suitability of these products, and their role in older IYC diets, is a concern. CPCF finger foods/snacks are typically rusks/biscuits, chips/crisps or melts, often mimicking the types of salty and sugary snack foods consumed by older children and adults, which many global and national guidelines discourage in older IYC feeding (National Health and Medical Research Council, 2012; US Department of Agriculture, & US Department of Health and Human Services, 2020; World Health Organization and the United Nations Children's Fund, 2021). While promoted for older IYC, CPCF finger foods/snacks often contain added sugar, high levels of total sugar and high levels of sodium (Bassetti et al., 2022; Maalouf et al., 2017). A recent assessment of CPCF products found that 28%–46% of CPCF finger foods available in Australia, the United Arab Emirates, the United Kingdom and the United States would warrant a red traffic light warning sign on their front‐of‐pack labels for high salt, sugar or fat content (Bassetti et al., 2023). The sweet and salty profile of many CPCF finger foods/snacks is problematic as it could establish unhealthy diet patterns lasting into childhood (Foterek et al., 2016). While in a HIC context high consumption of such products could contribute to overnutrition (Moore et al., 2019), in LMIC contexts—where nutrient‐density in older IYC diets is low—these products could also displace consumption of more nutrient‐dense options (Pries, Filteau, et al., 2019; Pries, Rehman, et al., 2019). As countries in Southeast Asia (SEA) face a triple burden of malnutrition, concurrent prevalence of undernutrition, overweight and obesity and micronutrient deficiencies (Rachmi et al., 2018), ensuring older IYC consume nutrient‐dense snacks without added sugar or excessive sodium content is critical.

Recent guidance from the WHO calls upon the use of nutrient profiling to establish thresholds to determine which CPCF products are high in fat, sugar and sodium and should not be promoted for older IYC (World Health Organization, 2017). While nutrient profiling models (NPM) have been developed in various regions and countries to evaluate the healthfulness of foods consumed by the general population, NPM specific to CPCF that take into consideration the unique nutrient requirements of this age group are scarce. In 2019, the WHO European Regional Office was the first institution to develop such a NPM (World Health Organization, 2019) and researchers have utilized this tool to assess products across the European region and to advocate for reformulation and stronger national regulations (Hutchinson et al., 2021). However, the use of this NPM to assess the suitability of CPCF products in other regions is limited. The genesis of such evidence in the SEA region could aid policymakers in the development and adoption of regulations to safeguard infant and young child nutrition. This study aimed to provide a comprehensive evaluation of the nutrient composition and labelling practices of CPCF finger foods/snacks in the market in seven SEA countries. The objectives were to: (1) determine the proportion of CPCF finger foods/snacks with inappropriate nutrient composition, (2) assess total sugar, sodium, total fat and saturated fat content of CPCF finger foods/snacks and (3) determine the proportion of CPCF finger foods/snacks with inappropriate labelling practices.

## 2 METHODS

### 2.1 Study design

For this study, a cross‐sectional assessment of label information was conducted among CPCF finger foods/snacks identified in the market in the capital cities of seven Southeast Asian countries: Phnom Penh, Cambodia; Jakarta, Indonesia; Vientiane, Lao People's Democratic Republic (PDR); Kuala Lumpur, Malaysia; Manila, Philippines; Bangkok, Thailand; and Hanoi, Vietnam. Label information was reviewed and compared against an adapted version of the WHO Europe NPM (adapted NPM CPCF) to benchmark products' nutrient composition and labelling practices against requirements. For this study, CPCF were defined as commercially produced foods or beverages specifically marketed as suitable for feeding children below 36 months of age, not including infant formula or other breast milk substitutes. CPCF identified in the seven countries had to meet at least one of the following criteria (World Health Organization, 2019): (1) recommended for introduction at an age of less than 3 years; (2) labelled with the words ‘baby’, ‘infant’, ‘toddler’, ‘young child’, or synonym; (3) labelled with an image of a child who appears to be younger than 3 years of age; or (4) in any other way presented as being suitable for children under the age of 3 years. The presence of images of bottles on labels or preparation instructions involving use of a bottle were considered to be ‘other ways’ a product may present itself as suitable for children under the age of 3 years. Within this overall category of CPCF, CPCF finger foods/snacks were defined as products that included confectionery items/sweet spreads/fruit chews, fruit finger foods/snacks (fresh or dry) and grain/starch based finger foods/snacks.

### 2.2 Product sampling and data extraction

In all seven capital cities, product sampling was conducted in August–November 2021. In Phnom Penh, Vientiane, Jakarta, Kuala Lumpur, Bangkok and Hanoi, a store scoping was conducted to generate a list retail outlets where CPCF were sold, and retailers were then sampled from this list First, an internet search of retail outlets in each capital city was performed, as well as discussions with local experts on which retail outlets were likely to sell CPCF. Following this, a compilation of larger retail outlets was prepared, and included supermarkets/hypermarkets, large grocery stores, large pharmacies and baby stores. Of those that were chain outlets, the largest physical stores were sampled along with all independent retail stores. In Kuala Lumpur, approximately half of all chain outlets (*n* = 21) and nine of the independent retail stores were purposively sampled due to the large number of retail outlets identified in the initial store scoping (*n* = 67). Online platforms for any of the sampled retail outlets were also included in the sample. Due to outbreaks of COVID‐19 during the period of product purchasing, only online retail platforms were visited for product purchasing in Jakarta, Kuala Lumpur, Bangkok and Hanoi; the sampled retail outlets in these countries that did not have online retail platforms were excluded. The final number of retail outlets visited in each location were: Phnom Penh = 28, Jakarta = 25, Kuala Lumpur = 30, Bangkok = 31, Hanoi = 21, Vientiane = 22. In each physical store/online retail platform, all shelves/search categories were reviewed and all unique CPCF products were sampled and purchased. Products were considered unique if they differed by brand name, subbrand name, descriptive name, age category/recommendation, manufacturer and/or flavour. Single serving and multi‐serving packages, different sizes of multi‐serving packages and bundles of single‐serving sachets/packages of the same product were considered a single product, as were products that differed only by the type of packaging.

For products in the Metropolitan Manila, a list of CPCF products previously identified during a 2020 study conducted in the Metropolitan Manila was reviewed (Bassetti et al., 2022). A search for these CPCF products was conducted through online retail platforms, and products were sampled and re‐purchased for this present study. National researchers searched websites for each product, matching the company, brand and product name before purchasing.

After products were purchased, they were photographed and label information was entered into a data set using the mobile app ONA Data app (www.ona.io). Double data entry of product label information was performed by two national researchers per country, and all inconsistencies reviewed and corrected. Double data entry took place in ONA Data app, where two national researchers entered the data independently. The data was then exported from ONA Data app into Microsoft Excel, and a comparison of the double data entry was conducted by a third researcher, and all inconsistencies reviewed and corrected. A 5% error check against the label images was implemented, and this check was repeated until the error rate was below 5%. National researchers first reviewed products to allocate them to one of six categories of the adapted NPM CPCF: (1) dry, powdered and instant cereal/starchy food (CPCF cereals), (2) soft–wet spoonable, ready‐ to‐eat foods, (3) wet meals with chunky pieces, (4) dry finger foods and snacks, (5) juices and other drinks and (6) other CPCF (World Health Organization, 2019). In this study, other types of CPCF included condiments, including powders, seasonings and flosses. Following product categorization, relevant label information for the assessment of product composition and labelling practices was extracted. This included: nutrient content declarations, serving size, ingredient lists and label text. Only label information in local languages or English was extracted. Products were excluded from the study if label information was not provided in the official local language of the country or English. In addition, duplicates of products purchased across retail outlets within the same country were removed from the data set. Extracted data in ONA Data app was exported as a Microsoft Excel database for analysis.

All national researchers who were involved in product purchased and data extraction received a standardized training. This training was provided remotely by the same technical team to ensure consistency. The training covered the definition of CPCF, the categories of CPCF, products that were not CPCF, and step‐wise instructions on how to enter label information into the ONA Data app.

### 2.3 Nutrient profiling and data analysis

The 2019 WHO Europe NPM was adapted for this study (World Health Organization, 2019), with this study's adapted model hereafter referred to as the ‘adapted NPM for CPCF’. Adaptation included several updates to align more closely with a finalized 2022 WHO Europe NPM (WHO Regional Office for Europe, 2022b) and an expansion of the nonpermitted claims details and categories. For this study, products within the ‘dry finger foods and snacks’ category were assessed against the adapted NPM for CPCF requirements. Within this category, there are three subcategories: (1) confectionery, sweet spreads and fruit chews, (2) fruit (fresh or dry), (3) other snacks and finger foods (including foods such as biscuits, rusks, chips/crisps, rice cakes, etc). If a product was categorized as ‘confectionery, sweet spreads and fruit chews’ it automatically failed the adapted NPM for CPCF because these products are considered de facto unsuitable for older IYC (World Health Organization, 2019). The remaining two subcategories were assessed against the adapted NPM for CPCF nutrient composition and labelling requirements.

The adapted NPM for CPCF has four nutrient composition requirements specific to CPCF finger foods/snacks: (1) no added sugar/sweetener (defined as mono‐ and disaccharides, syrups, nectars and honey, fruit juices and concentrated/powdered fruit juice (excluding lemon/lime juice), and nonsugar sweeteners), (2) <15% of total energy from total sugar (not applicable to products in the subcategory of ‘fruit (fresh or dry)’), (3) <50 mg sodium/100 kcal and <50 mg sodium/100 g, (4) ≤4.5 g total fat/100 kcal. The proportion of energy from fat was calculated using the Atwater factor system (the number of total fat g/100 g of product was multiplied by nine and then divided by total kcal/100 g of product). Where salt content was declared instead of sodium content, sodium content was calculated by dividing salt content by 2.5. Where products were missing the nutrient content information needed to assess the four nutrient composition requirements, the product was considered to have failed that requirement. Products had to have met all four requirements to pass the nutrient composition assessment.

The adapted NPM for CPCF has 12 labelling requirements for CPCF finger foods/snacks which cover the following aspects: (A) the protection and promotion of breastfeeding: (1) displays minimum recommendation of at least 6 months, (2) is not marketed as suitable for children less than 6 months of age through text or visual images, (3) contains a message on importance of continued breastfeeding to 2 years and beyond, (4) does not suggest superiority or equivalence to breast milk, (5) does not recommend or promote bottle feeding; (B) claims: (6) no nonpermitted compositional claims, (7) no nutrient content claims, (8) no nutrient function claims, (9) no disease risk reduction claims, (10) no other claims; and (C) product name and ingredient list clarity: (11) product name reflects ingredients in descending order as per ingredient list, (12) percentage of fruit stated in the ingredient list (pertains only to products containing fruit). Ingredient lists and label text were reviewed to assess products' performance against these 12 requirements. The presence of five categories of claims were assessed based on label text: (1) nonpermitted compositional claims, (2) nutrient content claims, (3) nutrient function claims, (4) disease risk reduction claims and (5) other claims. In line with the nutrient composition assessment, if a product was missing label information necessary to assess a labelling requirement, it failed that requirement. This was specifically relevant to the labelling requirements for age of introduction and ingredient list clarity; if a product presented no age of introduction or had no ingredient list/no percentages for ingredients requiring percentages, the product would fail that requirement. During data extraction, national researchers assessed labels and coded variables in the ONA Data App indicating whether products met the requirements or not. The national researchers were provided extensive training and a guidance manual, which provided detailed instructions on how to assess each requirement with notes and examples where relevant. By providing criteria for assessing these requirements, this subjective qualitative labelling assessment became objective and measurable.

Data analysis was conducted using Stata (version 14.2). Descriptive statistics were calculated, including proportions, frequencies and medians for non‐normally distributed data. The difference in adapted NPM performance of products manufactured internationally versus nationally was tested using the Pearson's *χ* ^2^ test, with significance defined as *p* < 0.05.

## 3 RESULTS

### 3.1 General characteristics of products

**Figure 1:**
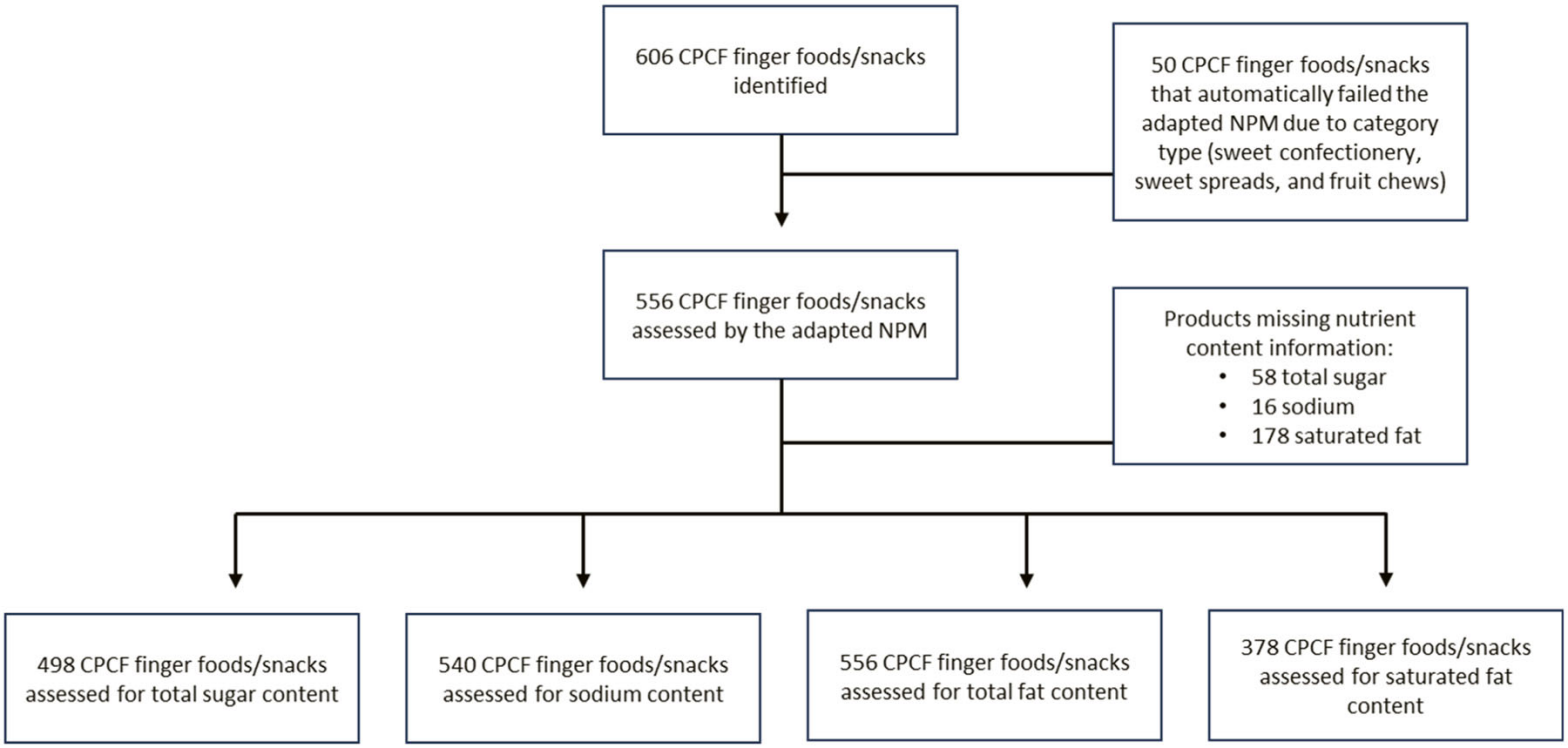
Product analysis flowchart: CPCF finger foods/snacks identified across seven Southeast Asia countries. CPCF, commercially produced complementary foods.

Of the 1635 CPCF products identified across all seven countries, over one‐third (37.1%, *n* = 606) were finger foods/snacks (Figure 1). This proportion varied across countries, with 37.0% (*n* = 84) of CPCF in Cambodia, 44.9% (*n* = 122) in Indonesia, 43.2% (*n* = 51) in Lao PDR, 45.4% (*n* = 176) in Malaysia, 23.6% (*n* = 43) in the Philippines, 42.2% (*n* = 87) in Thailand and 17.8% (*n* = 43) in Viet Nam classified as CPCF finger foods/snacks. Of all CPCF finger foods/snacks identified across the seven countries, 8.2% (*n* = 50) were ‘sweet confectionery, sweet spreads, and fruit chews’ and automatically categorized as not suitable for IYC. Fruit (fresh or dry) was a small proportion of the CPCF finger foods/snacks identified, accounting for only 1.3% (*n* = 8) and found only in Malaysia and Thailand (Table 1).

**Table 1:** Subcategories of commercially produced complementary food finger foods/snacks across countries.^a^

	Sweet confectionery, sweet spreads and fruit chews	Fruit (fresh or dry)	Other snacks and finger foods
Cambodia (*n* = 84)	13.1 (11)	—	86.9 (73)
Indonesia (*n* = 122)	6.6 (8)	—	93.4 (114)
Lao PDR (*n* = 51)	7.8 (6)	—	88.2 (45)
Malaysia (*n* = 176)	7.9 (14)	1.7 (3)	90.3 (159)
Philippines (*n* = 43)	14.0 (6)	—	86.0 (37)
Thailand (*n* = 87)	2.3 (2)	5.7 (5)	92.0 (80)
Viet Nam (*n* = 43)	7.0 (3)	—	93.0 (40)

^a^ Values are presented as % (*n*).

### 3.2 Nutrient composition of products

Of the 556 CPCF finger foods/snacks that were assessed against the adapted NPM for CPCF, only 14.8% (*n* = 82) met all relevant nutrient composition requirements (Table 2). Performance of CPCF finger foods/snacks varied across countries, with only 2.2% of products meeting nutrient composition requirements in Lao PDR versus 27.1% of products in Thailand. Across all CPCF finger foods/snacks, products achieved a median of two out of four nutrient composition requirements, with the requirement for no added sugar/sweeteners and the sodium requirement being the most common reason to fail. The presence of added sugar/sweeteners was particularly prevalent among products from the Philippines, Indonesia and Viet Nam, while over half of products in Thailand contained no added sugar/sweeteners. A similar trend by country was observed for the requirement to have a low percentage of energy from total sugar (under 15%)—only 30.0% and 40.5% of products from Viet Nam and the Philippines, respectively, met this requirement, as compared to 83.8% of products in Thailand. This observation mirrored the total sugar content of products (Table 3). The median total sugar content per 100 g was the highest in Indonesia, the Philippines and Vietnam, ranging from 14.1 to 14.3 g, while it was the lowest in Thailand at only 2.8 g. Median total sugar content per 100 kcal across all products was 3.0 g, close to the total sugar limit of 3.75 g per 100 kcal (15% energy from total sugar) set by the adapted NPM for CPCF. Less than half (44.4%) of all CPCF finger foods/snacks met the sodium requirement, with performance varying by country. Products from Lao PDR and Indonesia most commonly exceeded the maximum amount of sodium set by the adapted NPM for CPCF, with only one‐quarter (28.9%) and one‐third (33.3%) of products, respectively, containing <50 mg sodium/100 kcal and <50 mg sodium/100 g per product. Median sodium content was also the highest in Lao PDR and Indonesia, at 125 and 100 mg per 100 g product, respectively, as compared to 20–71 mg in the other countries. Median sodium content per 100 kcal across all products was 17 mg. Across all countries, the majority of CPCF finger foods/snacks met the total fat requirement, with this trending consistently across all seven countries. Median total fat content was generally low across all the countries; 8.7 g total fat per 100 g product and 2.4 g saturated fat per 100 g product among CPCF finger foods/snacks sold in Vietnam were the highest medians observed. The majority (70.3%, *n* = 391) of all CPCF finger foods/snacks were produced by international manufacturers headquartered outside the country where the products were sold. When comparing products from national manufacturers versus international manufacturers, there was no difference in the proportion that passed the nutrient composition assessment of the adapted NPM for CPCF (13.9% vs. 15.1%, respectively, *p* = 0.727).

**Table 2:** Proportion of CPCF finger foods/snacks meeting relevant adapted NPM for CPCF nutrient composition requirements.^a^

Country	*n*	No added sugar/sweetener^b^	<15% energy from sugar^c^	Met sodium requirement^d^	Met total fat requirement^e^	Met all relevant nutrient requirements	Median # of nutrient requirements met^c^
Cambodia	73	20.6 (15)	56.2 (41)	43.8 (32)	93.2 (68)	12.3 (9)	2 (0–4)
Indonesia	114	17.5 (20)	55.3 (63)	33.3 (38)	79.0 (90)	5.3 (6)	2 (1–4)
Lao PDR	45	24.4 (11)	86.7 (39)	28.9 (13)	86.7 (39)	2.2 (1)	3 (0–4)
Malaysia	162	32.1 (52)	54.7 (87)	56.8 (92)	95.1 (154)	22.8 (37)	2 (0–4)
Philippines	37	13.5 (5)	40.5 (15)	43.2 (16)	86.5 (32)	8.1 (3)	2 (0–4)
Thailand	85	51.8 (44)	83.8 (67)	44.7 (38)	90.6 (77)	27.1 (23)	3 (0–4)
Vietnam	40	17.5 (7)	30.0 (12)	45.0 (18)	95.0 (38)	7.5 (3)	2 (0–4)
All products	556	27.7 (154)	59.1 (324)	44.4 (247)	89.6 (498)	14.8 (82)	2 (0–4)

Abbreviation: CPCF, commercially produced complementary foods.

^a^ Values are presented as % (*n*) and median (range).

^b^ The following were considered added sugar/sweetener: sugar, juice (except lemon/lime), sucrose, dextrose, fructose, glucose, maltose, galactose, trehalose, syrup, nectar, honey, malted barley, malt extract, molasses.

^c^ Median and range presented. Not applicate to ‘Fruit (fresh or dry)’ snacks/finger foods (*n* = 3 in Malaysia; *n* = 5 in Thailand).

^d^ Requirement definition: Sodium <50 mg/100 kcal and <50 mg/100 g.

^e^ Requirement definition: Total fat ≤4.5 g/100 kcal.

**Table 3:** Median total sugar, sodium, total fat and saturated fat content per 100 g of commercially produced complementary food finger foods/snacks, by country.^a^

Country	Total sugar per 100 g (g)		Sodium per 100 g (mg)		Total fat per 100 g (g)		Saturated fat per 100 g (g)	
	*n*	Median [IQR]	*n*	Median [IQR]	*n*	Median [IQR]	*n*	Median [IQR]
Cambodia	64	12.4 [8.1–14.3]	73	71 [0–285]	73	0.0 [0.0–2.6]	55	0.0 [0.0–0.3]
Indonesia	111	14.3 [10.0–20.5]	114	100 [23–286]	114	10.0 [0.0–20.0]	49	0.0 [0.0–4.5]
Lao PDR	45	10.0 [2.8–12.4]	45	125 [25–179]	45	3.6 [0.0–8.3]	39	0.1 [0.0–1.4]
Malaysia	139	11.6 [2.3–18.8]	150	20 [7–180]	162	2.1 [0.7–11.0]	126	0.5 [0.1–2.0]
Philippines	30	14.1 [9.0–14.3]	37	71 [0–238]	37	0.0 [0.0–13.3]	27	0.0 [0.0–1.0]
Thailand	84	2.8 [0.0–13.3]	85	73 [0–160]	85	0.0 [0.0–8.0]	63	0.0 [0.0–1.4]
Vietnam	25	14.3 [12.5–16.0]	36	50 [12–313]	40	8.7 [1.3–11.1]	19	2.4 [1.4–3.6]
All products	498	12.4 [3.6–16.0]	540	71 [3–233]	556	2.7 [0.0–12.0]	378	0.2 [0.0–1.5]

Abbreviation: IQR, interquartile range.

^a^ Products without relevant nutrient content declarations on label are excluded.

### 3.3 Labelling practices of products

No CPCF finger food/snack product met all labelling requirements of the adapted NPM for CPCF (Table 4). Over half of all products across the seven countries (51.8%) met both labelling requirements related to product name and ingredient list clarity, however, performance varied across countries. While over half of products in most countries had product names that reflected their ingredient lists (with the order of foods in the name following the descending order in the ingredient lists) only one‐third (32.5%) of products in Vietnam and one‐fifth (20.0%) of products in Thailand met this requirement. While in most countries the majority of products presented the percentage of fruit content in their ingredient lists, this information was included in less than one‐third of products in Cambodia, Malaysia and the Philippines. Only one‐fifth (21.0%) of products across all countries met all five labelling requirements related to the protection and promotion of breastfeeding. This poor performance was primarily driven by products failing to have a message regarding the importance of continuing breastfeeding to 2 years of age and beyond. Malaysia was an exception to this finding with 80.3% of finger food/snacks meeting this requirement. Conversely, almost all products met the requirements of not suggesting superiority or equivalence to breast milk and did not recommend/promote bottle feeding. Approximately three‐quarters of all products provided a recommended minimum age of introduction of 6 months and did not market themselves as suitable for infants below 6 months of age. The presence of claims on product labels was the most common reason why CPCF finger foods/snacks failed the labelling requirements, with only 1.4% of all products meeting the five requirements related to claims. While the majority of products did not carry disease risk reduction claims or nutrient function claims, nearly half (48.7%) of all products carried a nutrient content claim (such as ‘Source of iron and B1’ or ‘Essential vitamins and minerals for babies’). In addition, the presence of nonpermitted compositional claims (such as ‘No added sugar or salt’ and ‘No preservatives’) and other claims (such as ‘Easy to grasp and hold’ and ‘Dissolves easily’) were identified on almost all products in Cambodia and the Philippines.

**Table 4:** Proportion of commercially produced complementary food finger foods/snacks meeting relevant adapted NPM for CPCF labelling requirements.^a^

	Cambodia (*n* = 73)	Indonesia (*n* = 114)	Lao PDR (*n* = 45)	Malaysia (*n* = 162)	Philippines (*n* = 37)	Thailand (*n* = 85)	Vietnam (*n* = 40)	All products (*n* = 556)
Protection and promotion of breastfeeding								
Minimum recommended age of introduction of at least 6 months	79.5 (58)	64.9 (74)	60.0 (27)	94.4 (153)	51.4 (19)	40.0 (34)	100.0 (40)	72.8 (405)
Not marketed as suitable for <6 months	90.4 (66)	71.1 (81)	97.8 (44)	59.9 (97)	67.6 (25)	98.8 (84)	75.0 (30)	76.8 (427)
Message on importance of breastfeeding ≥2 years	11.0 (8)	27.2 (31)	0.0 (0)	80.3 (130)	0.0 (0)	1.2 (1)	0.0 (0)	30.6 (170)
Does not suggest superiority or equivalence to breast milk	100.0 (73)	100.0 (114)	100.0 (45)	96.9 (157)	100.0 (37)	100.0 (85)	100.0 (40)	99.1 (551)
Does not recommend or promote bottle feeding	100.0 (73)	96.5 (110)	100.0 (45)	100.0 (162)	100.0 (37)	100.0 (85)	97.5 (39)	99.1 (551)
Subtotal	11.0 (8)	21.1 (24)	0.0 (0)	52.5 (85)	0.0 (0)	0.0 (0)	0.0 (0)	21.0 (117)
Claims								
No nonpermitted compositional claims	0.0 (0)	22.8 (26)	17.8 (8)	6.8 (11)	0.0 (0)	18.8 (16)	5.0 (2)	11.3 (63)
No nutrient content claims	50.7 (37)	29.0 (33)	64.4 (29)	56.2 (91)	48.7 (18)	54.1 (46)	42.5 (17)	48.7 (271)
No nutrient function claims	79.5 (58)	72.8 (83)	84.4 (38)	85.2 (138)	75.7 (28)	83.5 (71)	80.0 (32)	80.6 (448)
No disease risk reduction claims	93.2 (68)	98.3 (112)	100.0 (45)	100.0 (162)	100.0 (37)	100.0 (85)	100.0 (40)	98.7 (549)
No other claims	6.9 (5)	0.9 (1)	0.0 (0)	17.9 (29)	2.7 (1)	14.1 (12)	22.5 (9)	10.3 (57)
Subtotal	0.0 (0)	0.0 (0)	0.0 (0)	0.0 (0)	0.0 (0)	7.1 (6)	5.0 (2)	1.4 (8)
Product name and ingredient list clarity								
Product name reflects ingredients in descending order as per ingredient list	60.3 (44)	83.3 (95)	93.3 (42)	77.8 (126)	54.1 (20)	20.0 (17)	32.5 (13)	64.2 (357)
Percentage of fruit stated in ingredient list^b^	26.7 (12)	90.4 (47)	100.0 (30)	32.1 (26)	32.1 (9)	100.0 (57)	82.6 (19)	63.3 (200)
Subtotal	43.8 (32)	79.8 (91)	93.3 (42)	51.2 (83)	27.0 (10)	20.0 (17)	32.5 (13)	51.8 (288)
Met all relevant labelling standards	0.0 (0)	0.0 (0)	0.0 (0)	0.0 (0)	0.0 (0)	0.0 (0)	0.0 (0)	0.0 (0)

Abbreviations: CPCF, commercially produced complementary foods; NPM, nutrient profiling models.

^a^ Values are presented as % (*n*).

^b^ Question applicable to 316 products containing fruit (Cambodia *n* = 45; Indonesia *n* = 52; Laos *n* = 30; Malaysia *n* = 81; Philippines *n* = 28; Thailand *n* = 57; Vietnam *n* = 23).

## 4 DISCUSSION

Over one‐third of all CPCF products identified for sale across the seven SEA capital cities were CPCF finger foods/snacks, however, none were found to be suitable for promotion for older IYC according to the adapted NPM for CPCF. Of the total 606 products identified, 8.2% were automatically categorized as not suitable because they were confectionery items. Of the remaining 556 products assessed against the adapted NPM for CPCF, over 85% failed to meet all nutrient composition requirements, with the presence of added sugars/sweeteners, excessive sodium and total sugar contents the primary reasons for failure. Products also demonstrated concerning labelling practices, with almost all products (98.6%) displayed an inappropriate claim on the label. These findings reveal major concerns with the nutrient composition and labelling practices of CPCF finger foods/snacks in the SEA region and should serve as an alarm bell for regulatory action.

Global sales for CPCF finger foods/snacks have seen significant growth in the SEA region over the last decade—between 2011 and 2021, volume sales of CPCF finger foods/snacks grew by 78% and 50% in Indonesia and Malaysia, respectively, as compared to a growth of 22% and 17% for CPCF dry instant cereals (Euromonitor International, 2021). The presence of these products in the market is not unique to the SEA region, CPCF finger foods/snacks represent a sizable percentage of the market globally. An assessment of CPCF launched into the European market between 2017 and 2021 found that 18.3% were CPCF finger foods/snacks (Grammatikaki et al., 2021) and a separate assessment of CPCF products available in the market in 2016–2017 across 10 European countries found 12.9% to be CPCF finger foods/snacks (Hutchinson et al., 2021). In Asia, a 2022 assessment of CPCF products available in Japan found over one‐quarter (25.4%) were CPCF finger foods/snacks (Sugimoto et al., 2023). The growth of CPCF finger foods/snacks can be understood in the context of consumers' growing demand for convenience and reliance on packaged, ready‐to‐eat foods. A 2015 study in the United Kingdom found that most caregivers viewed CPCF products to be a convenient option when there was not time to prepare food (Maslin et al., 2015). A more recent study of British caregivers of older IYC revealed that CPCF finger foods/snacks are used to keep infants occupied while caregivers are busy with other tasks, and that CPCF snacks were perceived to facilitate independent eating by older IYC with minimal mess (Isaacs et al., 2022). Isaacs et al. note that selection of CPCF finger foods/snacks for child feeding was done primarily to meet the convenience needs of caregivers, rather than the nutritional value of these products, and it was therefore less important for caregivers that these products were healthy but more that they were not ‘actively unhealthy or unsafe’ (Isaacs et al., 2022). Convenience is a major driver of choice for consumers, including for caregivers of older IYC, that cannot be discounted.

Due to their health concerns, some types of CPCF finger foods/snacks should not be consumed by older IYC. Confectionery items, including candies and sweet spreads, have detrimental effects on oral health and are associated with increased risk of overweight and obesity throughout the lifespan. (World Health Organization, 2019). Across the seven countries in Southeast Asia, 50 confectionery CPCF finger foods/snacks were identified; all specifically promoted for consumption by older IYC. WHO Europe has called for unhealthy commercial food products intended for older children, including snack foods, energy drinks and sweet breakfast cereals, to clearly display a minimum age of 36 months/3 years on packages (WHO Regional Office for Europe, 2022a) to discourage consumption of these products among older IYC. While provision of snacks is an important part of ensuring adequate energy and nutrient intakes for older IYC, as well as increasing their exposure to varied textures and shapes of foods, it is critical that the foods provided as snacks are nutritionally appropriate for this age group.

While convenient, there are substantial concerns regarding the increasing availability of CPCF finger foods/snacks in the market. These foods have the potential to normalize consumption of packaged, highly processed snack foods early in life and could result in continued, adverse snacking habits throughout childhood (World Health Organization, 2019). The consumption of unhealthy sweet/salty food products has been shown to persist across the complementary feeding period in HIC and LMIC settings (Hinnouho et al., 2023; Rose et al., 2017), and has been associated with reduced nutrient intakes (Webb et al., 2006), poorer dietary adequacy (Pries, Rehman, et al., 2019) and an increased risk of overweight later in childhood (Rose et al., 2017). A study among children 9–15 months of age in Australia reported that CPCF finger foods/snacks were consumed with the same frequency as other snack food products aimed at older child/adult populations (Moore et al., 2022). Many of these products, including biscuits/rusks, puffs, melts and bites, mimic the form and function of snack foods targeted to older children and adults. They are promoted as being formulated for older IYC's specific dietary needs; however, they are often nutritionally similar to confectionery items. In the present study of CPCF across the SEA region, the median total sugar content among all CPCF finger foods/snacks was 12.4/100 g product and exceeded 14/100 g in three out of seven countries. Similarly, a study of CPCF available across Europe found mean total sugar content of CPCF biscuits/rusks and CPCF snacks to be 16.1 and 15.4 g per 100 g of products, respectively (Grammatikaki et al., 2021), and median total sugar content of CPCF finger foods/snacks in Australia has increased from 19.3 to 23.0 g per 100 g of product over the last two decades (Mccann, Russell, et al., 2022). As has been noted previously (Bassetti et al., 2022), this range of total sugar content of CPCF finger foods/snacks is similar to sugar content in biscuits/snacks for consumption among the general population, and Geraghty et al. observed that among CPCF biscuits available in Ireland, 67% had a greater total sugar content than digestive biscuits (Geraghty et al., 2018).

Nearly three‐quarters of CPCF finger foods/snacks in this study contained added sugar/sweetener and almost one‐third had high total sugar content that exceeded 15% of total energy. Inappropriate addition of sugars and high sugar content have been noted among these products in other contexts. An assessment of CPCF products across Europe found that among CPCF sweet snacks/confectionery items, the contribution of total sugar to total energy ranged from 23% in Spain to 44% in the United Kingdom, and exceeded 20% for CPCF rusks/biscuits in four out of six countries (Hutchinson et al., 2021), and a study of CPCF products available for online purchase in 2022 in Japan found that 84% of CPCF finger foods/snacks contained at least one added sugar (Sugimoto et al., 2023). The presence of added sugars and total sugar content of CPCF finger foods/snacks will vary by type of product within this category—a study of CPCF available in the United States found mean total sugar content of CPCF cereal bars/pastries, grain‐based desserts, dairy‐based desserts and savoury snacks to be 25, 12, 11 and 7 g per 100 g product, respectively, with 97%, 95%, 71% and 44% of these products containing an added sugar (Maalouf et al., 2017). As a comparison, in the United Kingdom, mean total sugar content of biscuits intended for general consumption (not CPCF biscuits) is 30 g per 100 g product (Hashem et al., 2020), indicating that some types of CPCF finger foods/snacks are similar in sugar content to snack foods consumed by adolescents and adults. In this same study, the majority of these sweet CPCF finger foods/snacks contained added sugars, along with the majority of savoury CPCF finger foods/snacks.

In addition to the concerning levels of sugar in the CPCF finger foods/snacks identified in the seven SEA countries, over half of products had high sodium content that exceeded the adapted NPM for CPCF requirement. High sodium content has been noted as a major concern in previous studies on the nutritional quality of CPCF. Sugimoto et al. reported that 41% of CPCF finger foods/snacks in Japan exceeded the Codex Alimentarius recommended limit of 200 mg sodium per 100 g product for these foods (Sugimoto et al., 2023) and a separate study found that 69% of CPCF finger foods/snacks available in the United States exceeded this limit (Maalouf et al., 2017). It is important to note that the sodium limit in Codex Alimentarius is four times higher than the 50 mg sodium per 100 g product requirement of the adapted NPM for CPCF, and therefore, far more lenient. Exposure to high levels of sugar and salt early in life can carry significant implications for childhood nutrition and can pose health implications later in life. Sugar intake during infancy has been shown to increase the risk of developing dental caries throughout childhood (Ruottinen et al., 2004), and high consumption of salt during infancy has been linked to high blood pressure among children (Genovesi et al., 2021). Preferences for both salty and sweet tastes during early childhood further influence consumption patterns throughout the lifespan (De Cosmi et al., 2017; Liem, 2017).

Product labels also revealed concerning practices, with nearly all labels displaying an inappropriate claim. The high prevalence of claims on CPCF finger foods/snacks has been noted previously among products available in HIC contexts (McCann et al., 2021; Santos et al., 2022), with some research showing an increase in the number of claims on CPCF labels (Mccann, Russell, et al., 2022). Claims are commonly used by manufacturers to influence consumer perceptions of a product, typically to increase perceived healthfulness or nutritional value, even among products that are of poor nutritional quality (Abrams et al., 2015; Andrews et al., 2000; Dixon et al., 2011). A study among Australian parents of toddlers found the likelihood of perceiving a CPCF finger food/snack as the healthiest option was nearly 14 times higher for products displaying a nutrition claim compared to those without a claim (Mccann, Woods, et al., 2022). A recent survey among caregivers of older IYC in the Southeast Asia region found that one of the most common reasons cited for purchasing CPCF products was the perceived health and nutrition benefits of products conveyed through product packaging (Walls et al., 2023.). Nonnutritive claims are also persuasive for caregivers motivated by claims related to child developmental milestones; a qualitative study among British parents found that claims such as ‘encourages self‐feeding’ reassured parents and facilitated perceptions of nonnutritive benefits among these products (Isaacs et al., 2022). Similar to many unhealthy products with added sugar/salt carrying nutrient content or function claims, CPCF finger foods/snacks claiming benefits for development are misleading. For example, many of the puffs/rusks/biscuits products are highly processed and extruded, causing them to melt when ingested by an older IYC, which can result in shortened oro‐sensory exposure time and disrupt experience of normal satiation cues (de Graaf, 2012). WHO Guidance on Ending the Inappropriate Promotion of Foods for Infants and Young Children states that use of health and nutrition claims on CPCF is inappropriate as they can mislead and confuse caregivers, as well as lead to inappropriate use of these products (World Health Organization, 2017).

This study is one of the first to comprehensively assess the nutrient composition and labelling practices of CPCF finger foods/snacks in the Southeast Asia region against international guidance. However, there are limitations. While the scoping of physical stores and online retailers allowed for an extensive sample of products available in each country, this scoping was limited to the capital cities and the sample achieved may not be representative of all products available nationally. Additionally, while not within the scope of this study, accuracy of declared nutrient content on labels was not verified through laboratory analysis. The assessment therefore relied on the information provided by manufacturers and actual nutrient content levels could differ.

## 5 CONCLUSION

CPCF finger foods/snacks comprised over one‐third of the CPCF products in the market in these seven Southeast Asian capital cities. Concerningly, the findings from this study reveal poor alignment of CPCF finger foods/snacks with both nutrient composition and labelling requirements of the adapted NPM for CPCF. While the majority of CPCF finger foods/snacks assessed contained added sugars and high levels of total sugar and sodium, many of the claims on the labels of these products presented the CPCF products as healthy and nutritionally appropriate options that can aid their child's development. These claims, along with increasing pressure on caregivers' time and need for convenience options for child feeding, are powerful influences for caregivers. Without strong, enforceable standards for both nutrient composition and labelling practices there is a serious risk that these foods can establish unhealthy diet patterns with risks for health and nutritional outcomes later in life. The commercial landscape of older IYC feeding has evolved rapidly in recent decades and regulatory and public health efforts must catch up to safeguard the health and nutrition of older IYC.

## AUTHOR CONTRIBUTIONS

Jessica Blankenship and Alissa M. Pries designed the study. Anzélle Mulder and Alissa M. Pries conducted the data management. Alissa M. Pries analysed the data. Eleonora Bassetti wrote the paper, and all authors provided substantial review.

## CONFLICT OF INTEREST STATEMENT

The authors declare no conflict of interest.

## Data Availability

The data that support the findings of this study are available from the corresponding author upon reasonable request.
